# Recent Advances and Applications in Starch for Intelligent Active Food Packaging: A Review

**DOI:** 10.3390/foods11182879

**Published:** 2022-09-16

**Authors:** Dandan Liu, Pei Zhao, Jinyu Chen, Yali Yan, Zijian Wu

**Affiliations:** 1 School of Biotechnology and Food Science, Tianjin University of Commerce, Tianjin 300134, China; 2 Tianjin Key Laboratory of Food Biotechnology, Tianjin University of Commerce, Tianjin 300134, China

**Keywords:** starch-based film, degradability, active packaging, intelligent packaging, mechanical properties, barrier properties, freshness indicator, time-temperature sensor

## Abstract

At present, the research and innovation of packaging materials are in a period of rapid development. Starch, a sustainable, low-cost, and abundant polymer, can develop environmentally friendly packaging alternatives, and it possesses outstanding degradability and reproducibility in terms of improving environmental issues and reducing oil resources. However, performance limitations, such as less mechanical strength and lower barrier properties, limit the application of starch in the packaging industry. The properties of starch-based films can be improved by modifying starch, adding reinforcing groups, or blending with other polymers. It is of significance to study starch as an active and intelligent packaging option for prolonging shelf life and monitoring the extent of food deterioration. This paper reviews the development of starch-based films, the current methods to enhance the mechanical and barrier properties of starch-based films, and the latest progress in starch-based activity, intelligent packaging, and food applications. The potential challenges and future development directions of starch-based films in the food industry are also discussed.

## 1. Introduction

From farm to table, food ingredients or products are susceptible to external damage or contamination, predominantly by pathogenic bacteria and oxidation, resulting in deterioration quality [1]. Therefore, adequate measures must be taken to maintain the original quality of the food. Among them, proper food packaging can maintain quality, avoid spoilage, extend shelf life, and reduce waste. In existing packaging materials, plastics are commonly used in food packaging due to their low price, good mechanical properties, and moderate barrier properties [2]. Each year, approximately 300 million tons of plastic are manufactured worldwide, and 40% are used in packaging materials [3]. Nonetheless, most plastic packaging is non-degradable [4] and causes severe environmental pollution [5]. Accordingly, the research and developing of environment-friendly food packaging to replace plastic packaging has gained increasing attention [6].

In recent years, degradable food packaging has developed rapidly. Diverse degradation technologies emerged with endless successions, such as photodegradable and biodegradable [7]. However, some degradable materials are toxic and hazardous to humans, and not all of them are suitable for food packaging [8]. Biodegradable materials, by contrast, are optimal for food packaging [9]. The function of biodegradable packaging film is the same as that of conventional packaging: to protect food quality, promote food circulation, and increase added value. At the end of the previous century, the focus of research switched from biodegradable film to completely degradable biofilms. To date, molecules used to assemble completely biodegradable films include polysaccharides (such as starch, chitosan, and cellulose) [10,11], protein (such as whey protein, soy protein, and silk protein) [12,13], lipids (such as beeswax and lauric acid) [14,15], and so on. The high cost of production and use limits the application of biodegradable materials in food packaging. Among the various natural polymers, starch is possible for food packaging because of its low price, abundant reserves, edibility, and degradability [16].

Starch is the primary carbohydrate storage form in plant tubers and seed endosperm, generally found in maize, potato, cassava, and cereals [17]. Starch is divided into amylose and amylopectin. The former is composed for α-1,4-glycosidic bonds connected end to end, which is a non-branching helical structure. The latter consists of α-1,4-glycosidic bonds, α-1,6-glycosidic bonds, which forms highly branched polymers with 24 to 30 glucose residues. Amylose content varies with different plant sources [18]. Due to the characteristics of organic starch itself (such as insolubility in cold water, hygrometry, poor structure, degradation, etc.), the application of natural starch in the industry is constrained [19]. In contrast, the mechanical properties of the pure starch-based film are considerably lower than those of conventional ordinary plastics [20]. In addition, starch molecules contain many hydroxyl groups, making them highly hydrophilic, resulting in poor water resistance and hydrophobicity and poor mechanical properties in wet environments [21,22]. Therefore, it is necessary to strengthen the mechanical and barrier properties of starch-based packaging materials.

Starch modification or blending with different materials is primarily used to resolve the above problems [23,24]. With the large-scale development of integrated technology in food processing, transportation, and storage, the food needs to have a long shelf life and maintain the quality of fresh food. The requirements for food packaging are also relatively increased [25]. The emerging active, intelligent food packaging can extend the shelf life of food, ensure food safety, and show information about food and its current status in the food supply chain to processors, retailers, and consumers [26,27]. Active packaging refers to the packaging system containing certain active substances (such as organic acids, enzymes, bacteriocins, natural plant extracts, etc.) [28], which can be released into the packaged food or the surrounding environment, thus extending the shelf life of food and retaining their quality, safety, and sensory properties [29]. Intelligent packaging is a system (including pH indicators and time or temperature sensors, etc.) that can monitor the storage status/cycle of packaged food or inform consumers about the quality of the food [30]. These intelligent packaging materials can broadly be pasted as labels or direct film formation [31].

In recent years, most of the studies on starch-based biodegradable films have focused on the sources of starch films [32], their processing methods [33], and the challenges and opportunities of starch-based materials [34]. Nevertheless, there is no current review on the status and application of starch-based biodegradable films in active, intelligent food packaging; this review first proposes the degradability of starch-based materials and methods to improve the mechanical and barrier properties of starch-based films. Then, the starch-based activity, the preparation of intelligent packaging for monitoring food quality, maintaining safety, and extending shelf life were evaluated, and the application of starch-based films as packaging materials in food preservation are analyzed. Finally, the existing problems are discussed, and the future research direction is adopted. It is of great significance and practical value to develop green, safe, and functional starched-based food packaging materials and their application in food preservation.

## 2. Starch-Based Biodegradable Film Materials

**Figure 1:**
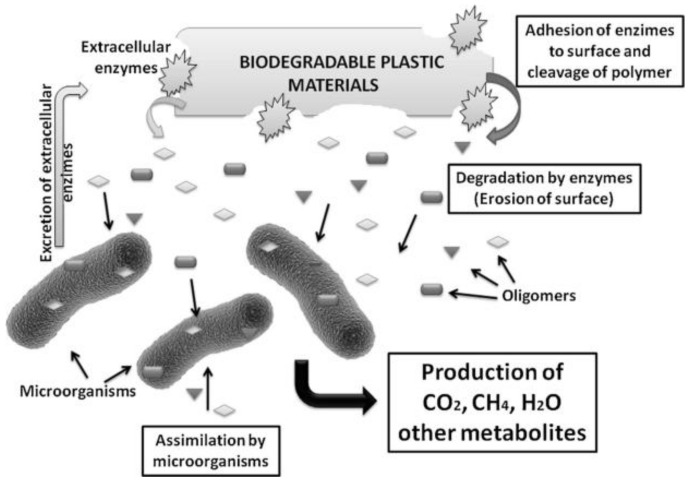
General mechanism of plastic biodegradation ref. [52]. 2019 Hesham Moustafa, Ahmed M. Youssef, Nabila A. Darwish, Ahmed I. Abou-Kandil.

Biodegradable plastics refer to those that can be degraded by indigenous micro-organisms under natural or unique conditions and eventually convert into environment-friendly biomass or small nontoxic molecule (such as CO_2_, CH_4_, or H_2_O) [35] (Figure 1). Among these, starch-based biodegradable materials have become one of the favored materials [36,37]. Starch is a kind of natural polymer compound belonging to the polysaccharide, which mainly exists in seeds, tubers, roots, or fruits of plants [38]. The raw materials used in starch manufacturing should first have the characteristics of high starch content and then have the characteristics of easy extraction, low processing cost, easy storage, and by-product production. Therefore, the main raw materials that meet the requirements are cereals, tubers, and legumes, which all contain a large amount of starch [39]. At present, starch extraction methods mainly include the alkali method, enzymatic method, and surfactant method. The Alkali method and enzymatic method use the action of alkali or enzymes to hydrolyze starch in combination with components, such as proteins and cellulose, and then release starch, improving the yield of starch; the surfactant method is to use sodium alkyl benzene sulfonate and other surfactants combined with protein so that the modified protein and starch form complex separation to achieve the purpose of starch extraction [40,41,42]. Starch properties vary by grain variety, growing climate, soil quality, and other growing conditions, ranging from more than 70% starch in cereals to 36% to 47% starch in dried beans. The content of vegetable starch is different. Potatoes account for approximately 14.7% and green leafy vegetables less than 0.2% [43,44].

Starch-based degradable packaging research and development began in the 1980s, and it experienced rapid growth during this period. After approximately 10 years of development, it demonstrated that only the starch component in those so-called degradable films could be degraded, while other film materials were just broken into fragments, which continued to exist in soil and water, still causing harm to the environment [45]. Hence, completely degraded materials were introduced at that time. Starch-based biodegradable materials are developed in three stages: Starch-filled plastics stage, Blended starch plastics stage, and All starch plastics stage. The increase in starch content has accompanied the evolution of starch-based plastics. Among them, all starch plastics can be completely biodegraded and have a comprehensive source of raw materials and low prices, which are the focus of starch-based material development.

1. Starch-filled plastics are made by mixing a small amount of original starch or modified starch with polyethylene or other thermoplastics and adding other applicable additives. Its purpose is to enhance the biodegradability of traditional petroleum-based starch materials. Nonetheless, its degradation still needs several years and cannot be thoroughly degraded [46];
2. Blended starch plastics are made of starch mixed with synthetic resin or other natural polymer materials. They are generally blends of starch/modified starch (30–60%) and synthetic biodegradable materials, which can be completely biodegradable and do not pollute the environment [47]. Compared with purely synthetic polymers, the blends degrade quickly and have better mechanical properties. Nonetheless, the added synthetic resins or other natural polymer materials are primarily polar compounds with hydrophilicity, and long-term exposure or contact with water will considerably degrade the properties of the plastic [48]. In addition, the compatibility between starch and additives, such as synthetic resins or other natural polymers is likewise problematic [49];
3. All starch plastics, also known as thermoplastic starch plastic, are a natural polymer biodegradable material. They are prepared by adding degradable plasticizers and other additives [50] through processes, such as extrusion, injection moulding, blow moulding, and calendering, which result in a “disordered” arrangement of starch molecule. The starch content of all starch plastics is above 90%, and a small number of different substances added as additives are nontoxic and can be completely degraded. Thus, all starchy plastics are genuinely and completely biodegradable. In addition, almost all plastic processing methods can be applied to all starch plastics [51].

The increasing use of starch-based biodegradable materials in food packaging has many advantages. These include reduced use of petroleum products, environmental friendliness and safety and reliability of food packaging [21]. Despite these advantages and benefits, biodegradable plastics currently account for less than 1% of total plastic production. Compared with traditional packaging materials, biodegradable materials have high production costs and poor mechanical and barrier properties, which are the main reasons for their limited application [53]. With the improvement of film material requirements, it will be very promising to develop antibacterial, antioxidant, and other multifunctional films based on improving the properties of the starch-based films.

## 3. Mechanical Properties of Starch-Based Films

Good mechanical properties are essential for the actual use of food packaging materials [54]. However, compared with similar applications of traditional plastics, the starch-based degradable packaging still has obvious disadvantages because of its poor mechanical properties [20], which limits its application in food and product packaging [22]. Excellent mechanical properties can be used to an impressive advantage in traditional packaging. Therefore, it is essential to strengthen the mechanical properties of starch-based films. Table 1 lists the mechanical properties of some starch-based films.

Studies have shown that the elongation of the starch film is negatively correlated with amylopectin content when starch is 6.3% to 25.0%. In contrast, the tensile strength of starch films was positively correlated with the amylose content, which was increased to 40% [62,63]. Hoang and Nguyen [64] studied cassava starch and mung bean starch with 26% and 33% amylose content, respectively, and found that the peak elongation and breaking elongation of mung bean starch film were 11% and 50% higher than those of cassava starch film, respectively. Paulina and Izabela [65] prepared films from starches isolated from pumpkin fruits, lentils, and quinoa seeds and compared them with potato starch films. The results showed that the tensile strength and elongation at the break of films are 8.98~13.85 MPa and 3.35~4.44%, respectively. All thin films are continuous elastic and have unlimited elastic behavior.

Although the pure starch-based film has mechanical properties that can be selected by researching the properties of various starches, it does not meet food packaging requirements. Currently, chemical and physical modification of starch is predominantly used to enhance the mechanical properties of the starch film [66,67]. Dai and Zhang [68] researched the impact of modification methods on the mechanical properties of starch-based films. The results indicated that the comprehensive properties of cross-linked cassava starch films were superior to that of other modified cassava starch films. The cross-linked modified starch molecules in the film increased the molecular weight of starch and expanded intermolecular interactions, resulting in better tensile strength. Adding cellulose nanofibers also improves the tensile strength and Young’s modulus of starch-based films [69]. The surface of cellulose particles contains many hydroxyl groups, which interact with starch molecules to form a dense network structure [70]. Ana and Buddhi [71] added lignocellulose into cassava starch film. Compared with the control group, the tensile stress of the film was 6.6 MPa (37.5% increased). The elongation at break was 44.43%, which was lower than that of the control group (54.92%).

In addition, starch can be acidified and hydrolyzed into starch nanocrystals to prepare enhanced mechanical properties of the material [72]. It has been reported that the natural crystals of starch do not improve the hardness and tensile strength of extruded film, while the plasticized starch obtained by recrystallization of V- and B-starch uses its compounding effect to increase tensile strength but reduce elongation at break [73]. Ren and Fu [74] hydrolyzed waxy corn starch with H6-sodium metaphosphate or glutaric acid to prepare cross-linked modified starch nanocrystals. The results showed that compared with the control group, the cross-linked starch nanocrystals improved the tensile strength and elongation at the break of starch film, but Young’s modulus remained unchanged.

## 4. Hydrophobic and Barrier Properties of Starch-Based Films

One of the main functions of food packaging is to maintain food’s stability and extend its storage period. In contrast, the barrier performance of packaging materials is a decisive factor affecting the shelf life of food [75]. Starch-based materials can permeate small molecules, such as gas and water vapor [76,77]. Accordingly, the barrier properties of starch-based degradable materials directly determine whether they can be used in food packaging.

### 4.1. Water Vapor Barrier

Spoilage of food is closely related to environmental humidity, and the water vapor barrier is critical for retaining or extending food shelf life [78]. Different foods have different requirements for a water vapor barrier. Dehydration should be avoided for fresh foods, but water vapor should be prevented for bread or cooked food [23]. Since starch is rich in hydroxyl groups and hydrophilic, the starch-based material is susceptible to water, and when it is combined with glycerol, the expansion of the network maintains a large amount of water. This expansion destroys the structural integrity of the matrix, leads to poor barrier performance, and cannot meet the packaging requirements of protective products, especially limiting the application scope of the film [79]. Researchers have added hydrophobic groups through starch modification to strengthen the hydrophobic properties of starch-based packaging materials. Hydrophilic hydroxyl groups in starch are esterified, etherified, crosslinked, and grafted with other substances to reduce the number of hydrophilic hydroxyl groups, thus enhancing the hydrophobic properties of starch [80,81]. Wongphan and Panrong [82] synthesized blended film with polybutylene adipate terephthalate (PBAT) by extrusion blending of protostars (NS), acetylated starch (AS), octenyl succinate starch (OS), and hydroxypropyl starch (HS). The results showed that the hydrophobic starch improved the compatibility and interaction with PBAT and greatly improved the barrier performance (82~89%). Nevertheless, modification of starch alone is insufficient to reinforce material properties.

In contrast, synergistic composite modification can increase the effectiveness of starch modification, thereby increasing the water resistance of starch-based materials. Cheng and Cui [30] researched the effects of natural and distinct binary changes [hydroxypropyl starch oxide, distarch acetyl phosphate, and oxidized acetyl starch] on the hydrophobic properties of intelligent films. The results demonstrated that the film of double-modified cassava starch had better water and water vapor resistance than the film of natural cassava starch.

In addition to directly modifying starch, it can be blended with some hydrophobic materials to improve the water-resistance of starch-based materials. Hydrophobic substances, such as lipids, hydrophobic nanoparticles, biomass materials, and their derivatives, are often added to starch films to strengthen their hydrophobicity. On the one hand lipids in the films can form a double layer by stacking hydrophobic lipids on a preformed starch-based film. On the other hand, lipid substances can also be added to starch film solutions in the form of emulsions to obtain starch-lipid composite films [83]. Bedroom [84] researched the effects of various lipids (oleic acid, palm oil, and margarine) and their concentrations on the water vapor permeability (WVP) of rice starch matrix film. The results showed that the addition of lipids reduced the WVP of rice starch matrix film. Compared with margarine and palm oil, the WVP value of oleic acid-doped films decreased with the oil content increase: Katiany and Adriana [85] mixed carboxymethyl cellulose with corn starch and cassava starch, respectively. The results showed that the WVP of the sample films was 48 and 40%, respectively. The interaction between starch and glycerol hydroxy and carboxymethyl cellulose carboxyl groups is supported by the high content of straight chain starch in the corn starch films and the high hydrophobicity. Mehran and Nahal [86] mixed Zataria multiflora Boiss (ZEO) or Mentha Pulegium (MEO) into a starch film by solution flow diffusion method. ZEO or MEO have enhanced the barrier properties of the starch film. It reduced the WVP by 50% compared to the control group. This may be due to the hydrogen and covalent interaction between the starch network and polyphenols, which lower the availability of hydrophilic groups and the affinity of the starch matrix for water molecules, leading to a decrease in the relationship of the film for water [87]. Some nanoparticles, such as ZnO nanoparticles (ZnO NPs), TiO_2_ NPs, SiO_2_ NPs, and nano-clay, have been demonstrated to be potent in enhancing water repellency. Zhang and Wang [88] studied the impact of SiO_2_ NPs with different particle sizes on the properties of potato starch film. The results showed that the addition of SiO_2_ NPs improved the water resistance of the film. Ni and Zhang [89] studied the adding of ZnO NPs into starch solution under an ultrasonic and magnetic angle and then flowed to form thin films. The contact Angle of the film increases from 85.73° to 121.45°.

### 4.2. Oxygen Barrier

Due to the growth of aerobic microorganisms and the chemical deterioration of food ingredients, the infiltration of oxygen dramatically affects the quality of packaged food [90]. To improve the barrier properties of starch-based materials to oxygen small molecules, appropriate fillers are usually added in the preparation process to form the composite material composed of starch matrix and stuffing to prepare the starch-based composites with high barrier properties. Compared with starch molecules, the structure of filler is more stable and denser. Small mass-transferring molecules, such as oxygen molecules, cannot directly transfer through, enhancing the barrier properties of composite films to small mass transfer molecules.

The combination of nanoparticles and starch has been proven to be an effective method to improve the oxygen barrier properties of starch-based materials. For example, Wattinee and Phatthtanit [91] used thermoplastic starch (TPS) as raw material to prepare composite starch films by extrusion and blow moulding using nirite and polybutylene diacrylate (PBAT). The results showed that nitrite modified the carbonyl group bond of PBAT, improved its compatibility with the TPS network, compacted its microstructure, and reduced the permeability of oxygen. Adding 5% nitrite to the PBAT/TPS blend system can effectively produce oxygen permeability similar to high-barrier fossil-based plastics. Pramod and Kalyani [92] prepared nanoclay-reinforced starch-polyacrylic acid hybrid nanocomposite films in an aqueous solution by in-situ polymerization technology. The results show that the oxygen resistance of starch-co-polyacrylic acid/clay film was significantly fortified during the loading process. Wang and Zhang [93] starch/polyvinyl alcohol/clay nanocomposite films were prepared by extrusion blow molding. Compared with starch/clay film, the oxygen permeability of starch /PVA/ clay film with 50% PVA content was reduced by approximately 210%. They also proposed a continuous phase change mechanism to explain the improved film properties. It is evident that starch/polyvinyl alcohol/clay nanocomposite films are a promising food packaging material with high barrier properties.

## 5. Starch-Based Active Films

Oxidation and microbial contamination are the leading causes of food spoilage. Starch packaging is similar to traditional packaging based on the problem of how to prolong the shelf life of products. It does not delay food spoilage by itself [94]. Food preservation is predominantly accomplished by adding antioxidants or antimicrobial substances to starch substrates to release active substances [95]. It can improve food quality and prolong food shelf life and has become a research focus [96]. In general, the antioxidant activity of film mainly depends on the potential release of active substances from the film matrix, which is closely related to the source of active substances, extraction conditions, additional amount, and the interaction between active substances and film matrix, and the microstructure of the film [97,98].

### 5.1. Antioxidant Active Starch-Based Films

The antioxidant active film can achieve an antioxidant effect by adding antioxidants to the packaging materials that can delay or prohibit food oxidation. When food is packaged in this packaging material, antioxidants are released into the interior of the packaging to extend the shelf life of the food and, to some extent, maintain the quality of the food [99]. Various antioxidants are used in food packaging and can be classified as naturally derived and synthetic sources. It is generally believed that chemically synthesized antioxidants have potential safety risks. Accordingly, naturally extracted antioxidants, such as essential oils and spices, have become the mainstream of current research [29]. Among them, absorption packaging by adding antioxidants to the packaging, absorption packaging of O_2_ to prevent food oxidation rancidity, packaging through the diffusion of antioxidants to the food surface or released into the packaging environment can inhibit its oxidation rancidity, so that the food is in a safer state. The fixed type can only keep the parts directly in contact with the package.

Oxidative degradation of foods is one of the significant non-microbial causes of food spoilage. Prevention of oxidation is most important for maintaining nutritional quality in foods, such as fresh produce, processed foods, and fresh meat [100]. The use of active film with antioxidant properties can inhibit the oxidative deterioration of food by adding active antioxidant substances during storage [101]. Various types of antioxidants can be classified as synthetic and natural antioxidants according to their sources.

Synthetic antioxidants, such as butyl-hydroxytoluene and butyl-hydroxyanisole (BHA) starch substrates to prevent lipid oxidation, have been commonly used in food packaging [102]. Nonetheless, the demand for natural antioxidants has lately expanded due to synthetic compounds’ potential toxicity and carcinogenicity [103]. Natural antioxidants, such as polyphenols, tocopherols, plant extracts, and essential oils, are preferred to be added to active packaging materials [104]. Kumar and Akhila [105] added 20% pineapple peel extract to polyvinyl alcohol (vinyl alcohol)-corn starch film, and the results showed that the control film had no antioxidant activity, the film containing 20% pineapple peel extracts had a DPPH scavenging activity of approximately 42% in the film. Dariusz and Waldemar [106] prepared oxidized potato films loaded with sodium ascorbate (SA); at 100 mM ascorbate ion concentration, the sample’s oxidation resistance activity and anti-free radical activity films were 7 times and 20 times higher than those of the control.

Due to their apparent volatility, volatile antioxidants in packaging materials delay food oxidation more effectively than non-volatile compounds. Essential oil, as a volatile antioxidant, is widely used in starch-based films to enhance their antioxidant properties. Elham and Majid [107] analyzed the antioxidant capacity of corn starch film supplemented with multi-flower corn essential oil and cinnamaldehyde in conventional, nano, and enhanced nanoemulsions. The results showed that the starch film containing nanoemulsion had higher antioxidant activity than the traditional sample film. In starch-based film materials, essential oil vapor diffuses into the internal atmosphere. It directly interacts with food, producing antioxidant protection [108]. These findings can be applied to the food packaging industry, especially meat and meat products susceptible to spoilage. Of course, the antioxidant effect of the starch films with essential oil nanoparticles in different food systems needs to be further research.

### 5.2. Antibacterial Active Starch-Based Films

The growth of microorganisms in food can lead to food spoilage, which reduces food’s nutritional value and safety [109]. As with traditional petroleum-based packaging, although modified starch-based films have good properties when applied to food packaging, they have weak antibacterial activity [110]. To resolve this problem, the antibacterial properties of the film can be accomplished through a hydrogen bond, electrostatic interaction, and other interactions between starch functional groups and antibacterial substances [111]. Compared to adding antimicrobial agents directly to food, making antimicrobial starch-based film prevents food spoilage by interacting with the active substance inside the package and the food. During this process, the active substance is slowly released around the food, effectively inhibiting the growth of bacteria. Depending on the user’s requirements, starch-based packaging materials can be satisfied by adding the appropriate active substance inside. The antibacterial agents added to the packaging film are generally classified natural antibacterial agents, inorganic antibacterial agents, and organic antibacterial agents.

Natural antibacterial materials mainly come from animals and plants, as well as microorganisms and their derivatives [112]. Common materials include essential plant oil, chitosan, antimicrobial peptides, etc. all of which have a bactericidal role: releasing bactericidal substances to change cell permeability and antagonize microorganisms [113,114]. Paola and Daniela [115] studied the fresh-keeping effect of rice starch film containing essential oregano oil on frozen fish. The results showed that the composite film containing oregano leaf essential oil had antibacterial activity compared with the control group. The shelf life of fish fillets packaged by the active film was prolonged. Cristina and Lorena [116] obtained cassava starch-chitosan films by melt bending and compression molding. The results showed that film could reduce the coliform group and total oxygen demand of frozen pork slices and prolong the shelf life of pork slices. Although there are more and more studies on starch-based natural antibacterial films due to their green, safe, broad-spectrum antibacterial properties and good biocompatibility, the problems of poor chemical stability and high extraction cost of raw antibacterial materials should also be considered.

Organic antimicrobial with some application are quaternary ammonium salts, polyphenols, pyridine, etc. They kill bacteria by electrostatic adsorption, have a powerful antibacterial effect, and are inexpensive [117]. However, organic antimicrobial agents have not received much attention because of their high toxicity and the tendency to produce drug-resistant bacteria in excessive use. Inorganic antibacterial agents primarily refer to metal antimicrobial agents. It is favored for its broad antimicrobial spectrum and excellent antimicrobial properties, which have become a leading research direction [118].

Inorganic antimicrobial agents generally refer to nano-metallic materials, such as Ag and Cu, or photocatalytic antimicrobial agents, such as TiO_2_ and ZnO, which have a solid binding ability with the active enzyme center of bacteria. Metal ions released by inorganic antibacterial materials in specific environments will either compound with nitrogen and oxygen in proteins after entering bacteria, or destroy the spatial conformation of protein molecules, inhibit DNA replication of cells, hinder the normal physiological functions of bacteria, and lead to bacterial death [119]. Hu and Jia [120] prepared composite films by incorporating chitosan nanoparticles in a modified starch matrix. The antibacterial activity of starch-based film was positively correlated with the loading of nanoparticles, and the antibacterial activity against Gram-positive *Staphylococcus aureus* was stronger than Gram-negative *Escherichia coli*. Chen and Li [121] prepared composite films by in-situ reduction using carboxymethyl cellulose (CMC) and starch as reductants and stabilizers. The results showed that ACS film had apparent antibacterial activity against *S. aureus* and *E. coli*. With the increase of AgNO_3_ solution concentration, the inhibitory effect of ACS film was greatly amplified.

### 5.3. Controlled Release Starch-Based Active Films

Commonly used antioxidant and antibacterial materials have good antioxidant and antibacterial effects [122]. However, if the release of the active substance is slow, resulting in insufficient concentration of the active substance, the food is prone to spoilage; when the release rate is accelerated, the concentration of the active substance is too high, leading to degradation or interaction with the food components. Therefore, current and future research is focused on regulating the release of active ingredients from films.

Controlled release packaging (CRP) extends product shelf life by controlling the release of active substances in food storage [123]. There are many ways to design CRP, generally including chemical modification, multilayer preparation, and cross-linkers. In recent years, microencapsulating active compounds and utilizing a metal-organic framework (MOF) in an active starch film matrix are the latest techniques for preparing CRP systems [124]. According to the mechanism of action, the controlled release system can be divided into a release system and an absorption system. In the release system, antimicrobial agents to the food surface as active agents to prevent food spoilage and quality loss [125]. Surfactants combine with starch matrix to form composite films, which are induced to release active substances through expansion, disintegration, diffusion, or disintegration [126]. The starch-based film belongs to the bottom-induced release type. Due to its moderate diffusion coefficient in the starch-based film system, the added active agent cannot diffuse in the starch matrix. Since most foods are humid and contain a lot of water, when the starch matrix is placed in a consonant liquid medium, the starch expands into the matrix through the water. In the expansion state, the diffusion coefficient of the active agent increases and then diffuses outwards [127]. In other words, the higher the moisture content of food, the higher the spoilage rate, and the higher the release rate of active substances in starch-based film, indicating that film can prolong the shelf life of products.

Zhang and Zhao [128] prepared active films using corn starch (CS) and zein rutin composite nanoparticles (RNs) as raw materials. The experimental results showed that the initial release rate was fast, and the cumulative release amount reached 19.8~27.1% after 2 h due to the weak binding or adsorption between RNs and CS. Rutin is released from CS film in the sustained release stage. After 12 h, the release of rutin was only 27.1~36.9% of the total rutin due to the migration of rutin from nanoparticles to solution. RNs dispersed in CS film can be controlled and released in aqueous food packaging. Farrag and Ide [129] prepared a starch film containing doughnut-like starch-quercetin particles, using pea and corn starch as raw materials. The in vitro release of quercetin film in aqueous ethanol was researched. The quercetin release of grain starch film reached equilibrium within 1 to 4 days, and that of legume starch film reached equilibrium over 1 week.

## 6. Starch-Based Intelligent Films

In addition to extending the shelf life of food through active substances, a new type of intelligent food packaging can be developed by giving new functions to starch-based packaging materials [130]. Intelligent packaging can monitor the quality of internally packaged food or detect the surrounding environment of food [131]. Natural active substances used in intelligent films usually have antibacterial and antioxidant activities. Consequently, in most cases, intelligent packaging is simultaneously active, but it is rarely studied to evaluate the two functions simultaneously [132].

Compared with other degradable polymers, the most significant advantage of starch-based film is that it is colorless and transparent, and the color change of food packaging film will not be affected by the sample matrix. Starch-based intelligent food packaging mainly combines indicators and provides intuitive, quantitative, or semi-quantitative information about packaged food through visual changes, such as color [133]. It includes explicitly freshness indicator (indicating the remaining shelf life of food by reacting with some characteristic gases generated in the storage process), time-temperature indicator (showing the remaining shelf life of food by time-temperature accumulation effect), etc. [134].

### 6.1. Freshness Indicator

The film-forming ability of starch makes the biopolymer an ideal proppant for preparing intelligent colorimetric films. Recently, interest in developing intelligent pH-sensitive films using starch has increased. pH changes are the primary food freshness and standard conditions. As food rots under the action of microorganisms, the pH value around food changes, so the relationship between food freshness or quality and pH value can be verified [135]. Organic pH indicators are not harmful to the human body or the environment. They come from a wide range of sources [136]. It is currently a popular topic of research in intelligent food packaging, including anthocyanins (ATH), curcumin (CR), and carotenoids, etc. [137]. Compared with other natural pH indicators, anthocyanins have a more comprehensive color range and a more significant color difference. The main methods of pH determination are colorimetry and electrochemical process.

Starch as a film matrix, mixed with indicators, can respond by sensing changes in food. When food rots, ammonia, dimethylamine, triathlon, and other gases will be produced. These gases will change the pH value around the food, and the natural pigment in the packaging film will change the color through its mechanism. For example, bok choy anthocyanins generally increase with pH, ranging from mauve to blue-purple to blue-green. This distinct color change allows the consumer to clearly identify whether the food is at the fresh, medium fresh, or spoiled stage and bring reference to consumers’ consumption. Choi and Lee [138] designed a colorimetric pH indicator film based on agar/potato starch/anthocyanins extracted from sweet potatoes. When the film is used as a pork package, the color shifted from red to green with the change of pH value and the deterioration of the sample. Mayra and José [133] studied the pH monitoring system of chitosan/corn starch/purple cabbage extract. They used it as a visual indicator of fish decay.

Electrochemical methods in pH sensors are used to convert chemical information into electrical signals for analytical experiments. The sensor receives chemical information and converts it into usable energy, which it converts into electrical signals [139]. In food packaging, chemical byproducts of spoiled food interact with the electrodes and begin to produce chemical changes. Compared with electrochemical sensors, the sensitivity of pH sensors is more strongly correlated with colorimetric sensors [140]. With colorimetric sensors, many visual perceptions come from the color intensity, sensitivity, or pH range. Increasing the proportion of pH-sensitive substances and decreasing the number of binding substrates can promote this increase in color intensity. The higher the anthocyanin content, the higher the color intensity; the more porous the starch content, the higher the sensitivity; the higher the cellulose binder content, the greater the mechanical strength of the sensor.

### 6.2. Time-Temperature Sensor

Perishable food is sensitive to temperature, and low-temperature storage can effectively prolong its shelf life. High temperatures accelerate the deterioration of food quality and lead to food reaching the end of shelf life in advance. Time and temperature are key factors affecting the quality of most foods. The time–temperature indicator (TTIs) can record and indicate the temperature change of the remaining shelf life of food during its circulation [141]. Through product time and temperature information, the temperature change of the product in each link can be monitored to ensure the quality and safety of food. This provides irreversible, visible color changes associated with temperature changes [142], which are caused by chemical changes [72], microbial changes [143], enzyme changes [144], or physical changes [145]. The range from activation to termination is usually reflected by color changes, corresponding to the shelf life of accompanying foods [146].

Compared to other areas, research on time and temperature indicators began late, and there is little relevant research. For example, Carolina and Pricila [147] added myoglobin extract and nitrite to the thermoplastic sensor film of cassava starch as an alternative to traditional electronic time-temperature sensors. They developed a natural, non-toxic, biodegradable thermochromic protein-based sensor. To investigate the color changes of myoglobin and myoglobin nitrite proteins under temperature. The sample film’s visual and instrument color changes in different environments demonstrate its feasibility as a time-temperature sensor for packaging or labels. Nogueira and Fakhouri [148] subjected starch-based edible films containing freeze-dried blackberry particles to sterilization at 127 °C for 15 min. The films underwent a significant change from red to brown color.

Starch films have made great progress in mechanical and barrier properties, and a large amount of research work on this subject shows the promise of starch films as an alternative to petroleum-based polymers as food packaging materials. Starch modification and additives have proved successful in producing films with similar properties to conventional packaging materials. In addition, starch-based films are used as carriers of functional ingredients to prepare active and smart packaging by combining antibacterial, antioxidant, and indicator agents to improve shelf life and quality, while facilitating the observation of food spoilage levels.

## 7. Starch-Based Active and Intelligent Films Application in the Food Industry

In response to consumer demand, there is a need to extend the shelf life of food products in the food industry. In the overall food circulation link, it is essential to maintain the high level of food quality. Certain starch-based biodegradable films have been used in food packaging. Table 2 lists their applications to some highly perishable, semi-perishable, and highly durable foods.

### 7.1. Active Packaging

**Figure 2:**
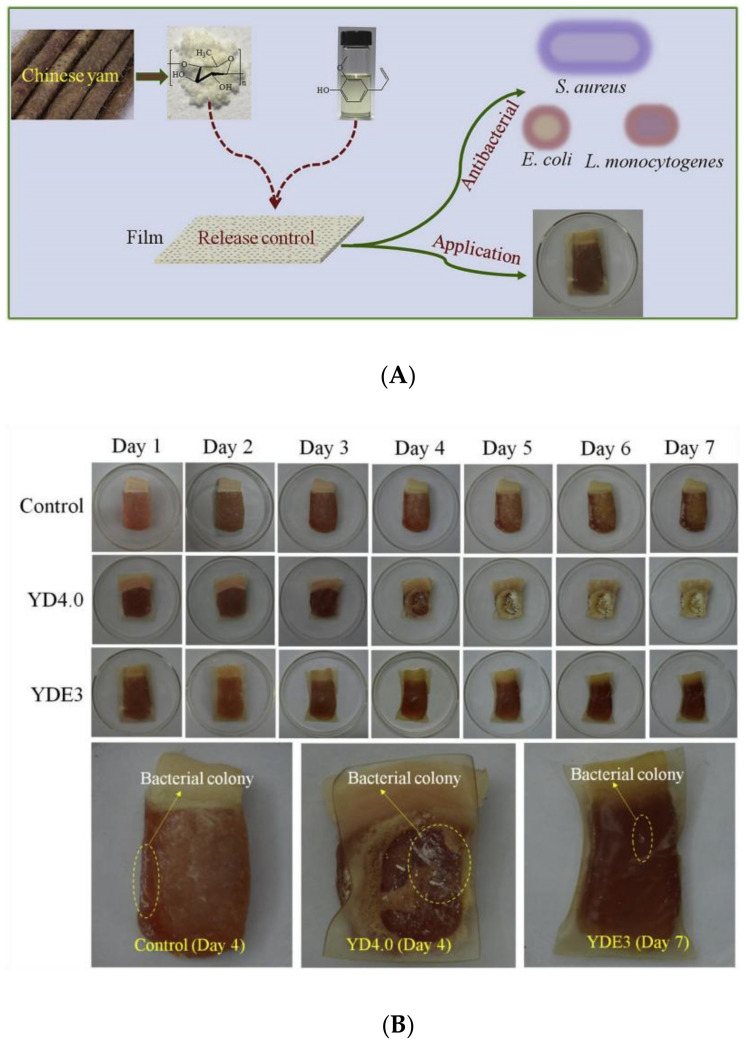
Application of active starch-based film (**A**) active packaging film based on yam starch with eugenol (**B**) application of antibacterial films to pork preservation. ref. [149]. 2019 Junfeng Cheng, Hualin Wang, Shaolei Kang, etc.

The application of starch-based active film in food packaging can effectively inhibit the growth of microorganisms and lipid oxidation in food, thus extending the shelf life of food [159]. This effect is mainly achieved through the antibacterial and antioxidant agent action of the film. When the film is used for food packaging, the active substances in the film can reach the food surface or the upper space of the packaging through diffusion, thus inhibiting the deterioration of food quality. Its preservation mechanism is ultimately the result of the interaction between active substances and food [160]. Currently, there is a growing number of studies on applying starch-based active film in food preservation. Cheng and Wang [149] prepared the starch-based antibacterial film for pork preservation using yam starch as a matrix and eugenol (YDE) (Figure 2A). The results showed that the antibacterial activity of YDE’s antibacterial activity against *E. coli* was superior to that of *Listeria monocytogenes* and *S. aureus*. The antibacterial activity of YDE3 film was better, which could prolong the shelf life of pork by more than 50% (Figure 2B). Studies have shown that the hydrophobic active substance of EO can attach to the cell surface of microorganisms and enter through plasma, plasma-binding enzyme, and other targets, resulting in cell wall rupture and leakage of intracellular substances [161]. In active packaging, EOs are embedded in a starch-based film, which allows for the bacteriostatic active compound to be released from the package longer, prolonging the time of food transportation and storage [162]. Thermoplastic starch/montmorillonite films containing EO components, such as thymol and carvacrol, were prepared and placed in PET containers to release EO as water vapor for the preservation of strawberries [163].

However, research into the application of starch-based active film in food packaging is still in the preliminary stage. There are still many problems worthy of further development, such as the antibacterial/antioxidant mechanism between starch film and food, the effect on food flavor, and other quality issues [164].

### 7.2. Intelligent Packaging

**Figure 3:**
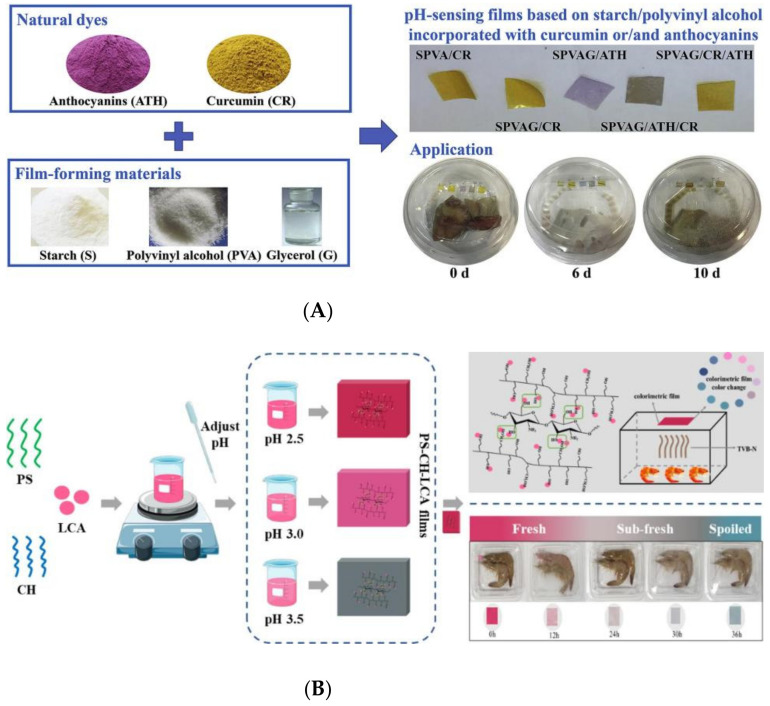
Application of Intelligent starch-based film (**A**) novel pH-sensitive films containing curcumin and anthocyanins to monitor fish freshness. (**B**) preparation and application of chitosan/starch based colorimetric film for sub-freshness monitoring. Ref. [164]. 2022 Bin Li, Yiwen Bao, etc. Ref. [165]. 2020 Hui-zhi Chen, Min Zhang, etc.

In addition to applied research on active packaging, scholars have also developed intelligent packaging film materials based on starch. At present, they are primarily focus on indicator intelligent packaging materials. Indicative intelligent packaging combines intelligent functions with standard packaging technology and provides consumers information through external color changes. Chen and Zhang [165] prepared visual pH-sensitive films containing (CR) and (ATH) as packaging indication labels for real-time non-destructive detection of fish freshness (Figure 3A). The results showed that starch film mixed with CR and ATH could provide three different colors: indicators of freshness, medium freshness, and spoilage of packaged fish. Using potato starch (PS), chitosan (CH), and floss lonicera anthocyanins (LCA) as raw materials, pH and NH_3_ response coloristic film (PS-CH-LCA) were prepared by controlling the pH value of the film-forming solution and applied to real-time monitoring of shrimp freshness (Figure 3B). The results showed that PS-CH-LCA (pH = 2.5) film was sensitive to color changes and highly correlated with spoilage indicators, indicating that the film could well reflect the freshness, sub-freshness, and spoilage degree of shrimp [166].

Based on the existing studies, intelligent packaging materials are mainly prepared using the color principle of CR and ATH, which has a good indicator effect on food with a significant change in pH value during food spoilage. Still, the precise relationship between pH value change and quality change remains to be further studied. In addition, the film material with antibacterial function has been prepared based on intelligent packaging, so developing intelligent, active packaging material is a research direction in the future.

## 8. Conclusions and Future Perspective

Starch-based biodegradable materials may play an important role in the future development of sustainable food packaging materials, reducing the energy and environmental stress of petroleum-based packaging materials. The current limitation is mainly due to the poor mechanical properties and barrier properties of starch-based packaging materials due to the properties of starch itself. Researchers can solve this problem by physically or chemically modifying starch or mixing it with other biopolymers and functional additives. After meeting the basic conditions of food packaging, starch-based active packaging can be prepared by adding antioxidant or anti-bacterial substances, which can extend the shelf life of food and reduce food waste. Starch time and temperature indicator films can detect food freshness in real-time by the color reaction. Starch-based films are widely used in food packaging and have a good protective effect on fresh food. In the future, it will be possible to prepare active or smart packaging using starch-based materials.

Although some of these methods have improved the properties of starch-based films, more research is still needed to create starch-based films with similar mechanical and barrier properties to traditional plastic packaging. Starch-based biodegradable materials can be affected by starch type, preparation techniques, storage conditions and other factors during preparation, resulting in a lack of homogeneity and stability of the product. Currently, most research on food packaging applications is completed at the laboratory level. Industrial manufacturing, safety regulations, environmental issues, and consumer acceptance have also limited the commercialization of starch-based films. In addition, the combination of starch and other materials needs further study to meet practical needs. Many additives have great potential in developing antibacterial and antioxidant films. There is still quite a lot of interesting work to be done in terms of developing antimicrobial agents that can be used in starch matrices. There is a lack of appropriate criteria for assessing and quantifying antimicrobial and antioxidant activity. For example, in “food mimics” solutions, the mimics corresponding to different foods should be expanded rather than limited to water. The field of starch-based smart films has great potential, although so far its functionality has been relatively limited. At present, pH indicators are mostly used in intelligent packaging. While changing the environment around the food in package, active substances will also change the pH change of the system, which may lead to the irreversibility or timeliness of indicators in packaging film and cause consumers to make a wrong judgment on product quality. Further studies are needed to investigate the combination of functional food components with multireactive starch membranes. By mixing with heat-sensitive, moisture-sensitive, gas-sensitive, and other multifunctional packaging materials to prepare new intelligent packaging, the film can have a variety of different functions, can prepare the corresponding stimulus response packaging according to the food needs, and have a slow/controlled release effect on the active substances in the film.

In summary, although starch-based active and intelligent packaging still has shortcomings in terms of material selection, preparation, and its role, there is no doubt that starch has become the most likely substrate to replace petroleum-based traditional packaging due to its structural properties and other advantages, and it has the possibility of substrates leading the development of intelligent food packaging in the future.

## Figures and Tables

**Table 1:** Mechanical properties of starch-based films.

Films	Additives	Thickness (mm)	Moisture Content (%)	Tensile Strength (MPa)	Elongation(%)	References
Cassava starch Mungbean starch Cassava: Mungbean (50:50)	glycerol	0.103	19.22	2.85	18.82	[55]
		0.098	19.66	9.34	21.37	
		0.090	22.11	7.93	21.32	
	sorbitol	0.101	9.43	6.77	14.86	
		0.113	9.16	19.20	12.89	
		0.105	8.84	15.87	10.84	
Wheat	glycerol	0.074	44.5	3.29	15.21	[56]
Corn		0.112	36.7	3.72	19.13	
Potato		0.055	31.6	6.56	5.67	
PV:PB (50:50, 60:40, 70:30, 80:20, 90:10, 100:0)		0.061~0.070	-	27.5~52.6	108.1~241.8	[57]
NF		0.064	13.06	3.49	19.21	[58]
ACT (4%, 8%)		0.071, 0.072	14.47, 13.43	3.69, 2.86	31.4, 19.5	
HPS (10%, 30%)		0.070, 0.067	16.49, 18.82	3.10, 2.54	57.17, 64.81	
Cassava starch				5.5	45.5	[59]
	(5~15) wt% metakaolin+ glycerol			5.7~8.1	23.1~33.2	
Rice starch	sorbitol			10.75	7.56	[60]
	(10~50) % NaOH+ sorbitol			2.75~9.87	11.36~53.03	
Octenyl succinate starch	glycerol	0.087	29.54	9.60	32.41	[61]
	(0.025~0.100) % PSE+ glycerol	0.090~0.091	29.22~29.62	7.56~8.62	23.99~30.98	
	(0.025~0.100) % HSE+ glycerol	0.090~0.091	29.74~30.03	7.31~10.58	29.58~31.65	

PB: Pinto Bean Starch, PV: Polyvinyl Alcohol. NF: native starch; PSE: pecan nutshell extract, HSE: hazelnut skin extract.

**Table 2:** Highlights for applications of starch-based films on food products.

Starch	Additives	Product	Finding	References
Yam starch	eugenol	pork preservation	with 3% eugenol can extend the shelf-life of pork beyond 50%	[149]
Job’s tears starch	clove bud essential oil	pork belly	with 0.5% CBEO can reduce Lipid oxidation	[150]
Potato starch	carrot anthocyanins	row milk	used as an indicator to monitor freshness/spoilage of milk	[151]
Cassava starch	gelatin and casein	guavas	increased the guavas shelf-life by 2 days	[152]
Maize starch	grape juice	chicken breast fillets	delayed the lipid oxidation and microbiological growth of chicken breast fillets.	[153]
Brazilian pine seed starch	citric pectin and functionalized	grapes and bread	maintained the quality for 30 days of storage	[154]
Hydroxypropyl distarch phosphate	ε-polylysine and gelatin	fresh bread	delayed microbial spoilage	[95]
Corn starch	carboxymethyl cellulose	food simulant	excellent antimicrobial activity towards *E. coli.*	[155]
Potato starch	betacyanin	fish	visual change from pink to yellow color of the package label paralleled the increase in total volatile base nitrogen (TVB-N)	[156]
Corn starch	curcumin-loaded Pickering emulsion	fish	the color of films changed from yellow to red	[157]
Cassava starch	lycium ruthenicum anthocyanins-loaded nano-complexes	micropterus salmoides	when the fillet of perch deteriorates, the film shows significant color change	[158]

## Data Availability

Data is contained within the article.

## Abbreviations

PB	Pinto Bean Starch
PV	Polyvinyl Alcohol
NF	native starch
PSE	pecan nutshell extract
HSE	hazelnut skin extract
WVP	water vapor permeability
CR	curcumin
ATH	anthocyanin
